# Generation of Recombinant Antibodies to Rat GABA_A_ Receptor Subunits by Affinity Selection on Synthetic Peptides

**DOI:** 10.1371/journal.pone.0087964

**Published:** 2014-02-19

**Authors:** Sujatha P. Koduvayur, Hélène A. Gussin, Rajni Parthasarathy, Zengping Hao, Brian K. Kay, David R. Pepperberg

**Affiliations:** 1 Department of Biological Sciences, University of Illinois at Chicago, Chicago, Illinois, United States of America; 2 Lions of Illinois Eye Research Institute, Department of Ophthalmology and Visual Sciences, Illinois Eye and Ear Infirmary, University of Illinois at Chicago, Chicago, Illinois, United States of America; 3 Department of Bioengineering, University of Illinois at Chicago, Chicago, Illinois, United States of America; Naval Research Laboratory, United States of America

## Abstract

The abundance and physiological importance of GABA_A_ receptors in the central nervous system make this neurotransmitter receptor an attractive target for localizing diagnostic and therapeutic biomolecules. GABA_A_ receptors are expressed within the retina and mediate synaptic signaling at multiple stages of the visual process. To generate monoclonal affinity reagents that can specifically recognize GABA_A_ receptor subunits, we screened two bacteriophage M13 libraries, which displayed human scFvs, by affinity selection with synthetic peptides predicted to correspond to extracellular regions of the rat α1 and β2 GABA_A_ subunits. We isolated three anti-β2 and one anti-α1 subunit specific scFvs. Fluorescence polarization measurements revealed all four scFvs to have low micromolar affinities with their cognate peptide targets. The scFvs were capable of detecting fully folded GABA_A_ receptors heterologously expressed by *Xenopus laevis* oocytes, while preserving ligand-gated channel activity. Moreover, A10, the anti-α1 subunit-specific scFv, was capable of detecting native GABA_A_ receptors in the mouse retina, as observed by immunofluorescence staining. In order to improve their apparent affinity via avidity, we dimerized the A10 scFv by fusing it to the Fc portion of the IgG. The resulting scFv-Fc construct had a K_d_ of ∼26 nM, which corresponds to an approximately 135-fold improvement in binding, and a lower detection limit in dot blots, compared to the monomeric scFv. These results strongly support the use of peptides as targets for generating affinity reagents to membrane proteins and encourage investigation of molecular conjugates that use scFvs as anchoring components to localize reagents of interest at GABA_A_ receptors of retina and other neural tissues, for studies of receptor activation and subunit structure.

## Introduction

GABA receptors, which bind the neurotransmitter γ-aminobutyric acid (GABA) [1], consist of two main families: GABA_A_ and GABA_B_ (GABA_C_ are a subtype of GABA_A_) [2]. GABA_A_ receptors are pentameric, ligand-gated chloride channels, and consist of varying combinations of 19 subunits (α1–6, β1–3, γ1–3, δ, ε, θ, π, ρ1–3). This multiplicity of subunits results in high GABA_A_ receptor diversity with respect to the cellular localization of these receptors and their developmental, physiological and pharmacological properties [3], [4], [5], [6], [7]. GABA_A_ receptors are expressed within the retina [8], [9], [10], [11], [12], [13], where they mediate synaptic signaling at multiple stages of the visual process [14], [15]. The abundance and physiological importance of GABA_A_ receptors in the central nervous system (CNS) [16] make this class of neurotransmitter receptor an attractive target for therapeutic design.

Traditionally, functioning of membrane receptors and effects of membrane protein activation is studied using monoclonal or polyclonal antibodies. However, generating these antibodies is a tedious, time consuming [17] and often-unsuccessful process. As an alternative, engineered antibody fragments show great promise as affinity reagents against membrane proteins [18], with single-chain variable fragments (scFv) of immunoglobulins being the most popular [19], [20], [21], [22]. ScFvs are 26–28 kilodaltons (kDa) in size and contain the variable domain of the immunoglobulin heavy and light chains, which are joined by a peptide linker that is often Glycine-Serine-rich [23]. ScFvs can be expressed as monomers or multimers, depending on the size of the linker [24], or through conjugation to dimerization domains such as leucine zippers [25]. As recombinant entities, their affinity and specificity can be engineered [26], [27], [28]. ScFv conjugates offer a higher rate of tissue penetration, than IgGs, but lower clearance level, than unconjugated scFvs alone, making them well suited for therapeutics and drug delivery [29]. ScFvs are also easily amenable to molecular manipulation for expression with fluorescent tags, which can be used to track their targets in a cellular environment. There are various methods of generating scFvs [30] from *in vitro* panning strategies on immobilized surfaces [31], [32], [33], [34] to panning with *in vivo* epitopes [35]. Given the ease of generation, ability for affinity maturation and decoration with fluorescent tags, scFvs provide a viable, cost-effective alternative to antibodies. Traditionally, using membrane proteins as targets for selections has been problematic, in part due to the difficulty in expressing and purifying native protein that retains its structure and function [36], [37], [38]. However, scFvs against membrane proteins can be generated to peptide fragments of these proteins, thereby overcoming the need for generating purified protein targets [39].

Previous studies have described the engineering of scFv-based antibody fragments as affinity reagents for neurotransmitter receptors [40], [41]. However, targeting specifically of GABA_A_ receptors by scFv-based reagents has not been extensively explored. In undertaking the present study, we reasoned that such an scFv construct could prove desirable for investigational and potentially clinical applications aimed at localizing GABA_A_-reactive pharmacological agents at the GABA_A_ ectodomain. For example, Yue et al. [42] have recently shown that “MPC088”, a photo-isomerizable derivative of the GABA_A_ modulator propofol (2,6-diisopropylphenol), exhibits pronounced light-dependent potentiating and direct agonist activity at GABA_A_ receptors (also see [43], [44]). Thus, an scFv-based anchor that binds to a specific GABA_A_ subunit, upon chemical conjugation with (otherwise diffusible) MPC088, could localize and thereby enhance MPC088's efficacy as well as targeting specificity at native receptors, avoiding the need for genetically engineered expression of a non-native receptor that tethers the photo-regulating ligand [45], [46], [47]. With the aim of developing an anchoring moiety for MPC088 and potentially other GABA_A_ receptor modulators that can be introduced into the eye via intra-vitreal injection, we undertook the present study to identify scFvs with affinity for the GABA_A_ α1 and β2 subunits. Our interest in propofol based modulators of GABA_A_ [42] and the role of GABA_A_ α1 and β2 in the binding of propofol [44], [48], [49], led to our objective of targeting these receptor subunits in the vicinity of the propofol binding sites. Our approach involved screening phage-displayed scFv libraries with synthetic peptides predicted to correspond to extracellular regions of the α1 and β2 subunits. Here we report the isolation of four scFvs (one against α1 and 3 against β2) and their expression as soluble proteins in the methanotropic yeast, *Pichia pastoris*. Equilibrium dissociation constants for these scFvs were in the low micromolar range as measured by fluorescence polarization (FP) and with the SRU BIND® System [50]. These results, together with fluorescence visualization data, obtained from GABA_A_ receptor-expressing *Xenopus laevis* oocytes, and immunocytochemical data, from rat retina obtained for one of the identified scFvs (anti-α1 scFv, A10), provide evidence that these peptide-raised scFvs specifically target α1 and β2 containing GABA_A_ receptors. In addition, electrophysiological data obtained from GABA_A_-expressing oocytes are consistent with the absence of an effect of this binding on channel activity. We have also expressed a dimeric, scFv-Fc version of A10, which has an improved binding (K_d_ = 26 nM) and higher detection limit compared to the monomeric scFv in dot blots. These scFvs and their dimeric versions can thus be used in various biochemical and cellular assays to determine GABA_A_ receptor expression, activity, and signaling

## Materials and Methods

### 2.1. Ethics Statement

All procedures involving experimental animals were conducted in accordance with institutional policies (Animal Care Committee of the University of Illinois at Chicago; Approved protocols 10–159 and 13–125), and with the Statement for the Use of Animals in Ophthalmic and Vision Research adopted by the Association for Research in Vision and Ophthalmology. Oocyte harvest surgery on *Xenopus laevis* (see section 2.9.1) was performed under MS-222 anesthesia. Mouse euthanasia (see section 2.9.2) was performed under ketamine/xylazine anesthesia.

### 2.2. Peptides, Plasmids, and Primers

Peptide sequences, corresponding to predicted extracellular regions of the α1 (QPSQDELKDNTTVFT) and β2 (MWRVRKRGYFGIWSFPLII) subunits, and the negative control peptides GBB5 (KLHDVELHQVAERV) and Pep1 (LRWFSHSTR), were chemically synthesized at the Research Resources Center of the University of Illinois at Chicago. These negative control peptides were chosen for their similarity in size (molecular weight) and sequence length to the GABA_A_ peptides. Moreover, these peptides are derived from reported retinal proteins (Pep 1 and GBB5). All of the peptides, except for those used for fluorescence polarization (FP) experiments, were biotinylated at their *N*-termini, amidated at their *C*-termini, and included a Serine-Glycine-Serine linker between the *N*-terminal biotin and peptide. Peptides that were used in FP measurements carried a fluorescein isothiocyanate (FITC) at their *N*-termini. FITC-labeled peptides were purified by high-performance liquid chromatography (HPLC). The maltose-binding protein (MBP) fusion peptides were generated by the Quikgene method [51], using the oligonucleotide primers described below.

In this study, the phagemid vectors pAPIII6 [52] and pSANG4 [53], and the expression vectors, *P. pastoris*, pPICZαC′, *E. coli*, pAT224, and mammalian pBIOCAM5 were used. The pPICZαC′ vector was modified from its parent plasmid pPICZαC (Life Technologies, Carlsbad, CA) by swapping the c-myc (EQKLISEEDL) epitope with the Flag (DYKDDDDK) epitope, and altering the multiple cloning site to include *Hind* III, *Sal* I, *Nco* I and *Not* I restriction endonuclease sites. The pAT224 (GenBank accession number AY327139) vector contains an Avitag at the N-terminus for *in vitro* biotinylation of MBP fusion constructs with BirA [54], and was a generous gift from Dr. Andreas Plückthun (University of Zurich, Switzerland) The pBIOCAM5 expression vector, a generous gift from Dr. John McCafferty (University of Cambridge, UK), contains the Fc portion of the human IgG molecule followed by three tandem Flag epitopes and 6× His tags, as well as a CMV promoter for extracellular expression in mammalian cell lines [55].

Oligonucleotides used for DNA sequencing are as follows (5′→3′): AOX1, GACTGGTTCCAATTGACAAGC; PicRv, GCAAATGGCATTCTGACATCC; for pPICZαC′ vector; M13LeadSeq, AAATTATTATTCGCAATTCCTTTGGTTGTTCCT; Notmyc, GGCCCCATTCAGATCCTCTTCTGAGATGAG; for pSANG4 vector, OmpAUp, CTGTCATAAAGTTGTCACGGCCGA for pAPIII6 vector; pAT224_FW primer1, TACTGCGGTGATCAACGCC; pAT224_REVprimer, CGTAACAAATCCAGATGGAGTTCTGAGG for pAT224 vector and upscFv-for, ATGAGTTGGAGCTGTATCATCCTCTT; dwnscFv-rev, TTCAGGATCTTCGTGGGACACGTCC; for pBIOCAM5 vector. Quikgene primers used for cloning α1 and β2 peptide sequences are (5′→3′): alpha 1 QG F, GAACTGAAAGACAACACCACCGTTTTCACCCAAGCTTCTCATCACCATCACCATCACTAAT; alpha1 QG R, GTTGTCTTTCAGTTCGTCCTGAGACGGCTGGGATCCAGTCTGCGCGTCTTTCAGGGCTTC; beta 2 QGF, GGTTACTTCGGTATCTGGTCTTTCCCGCTGATCATCCAAGCTTCTCATCACCATCACCATCACTAAT; beta2 QGR, GATACCGAAGTAACCACGTTTACGAACACGCCACATGGATCCAGTCTGCGCGTCTTTCAGGGCTTC. Cloning primers for A10 (5′→3′): pBIOCAM5-A10 UP, CAGGCGCCATGGATAAGCTTTCCTATGAGCTGACT and pBIOCAM5-A10 rev, GTCACCGTCTCCTCAGTCGACGCGGCCGCAGACAAGACC.

### 2.3. Affinity Selection

For isolating subunit-specific binders, three rounds of affinity selection were performed with biotinylated α1 and β2 peptides and two recombinant human phage-displayed libraries [52], [56]. The biotinylated peptides (200 µL, 10 µg/mL) were captured on the surfaces of Nunc polystyrene microtiter plate wells (Thermo Fisher Scientific, Pittsburgh, PA) via NeutrAvidin™ (200 µL, 10 µg/mL; Thermo Fisher Scientific), and non-specific binding sites were subsequently blocked with 2% skim milk in phosphate buffered saline (PBS: 137 mM NaCl, 3 mM KCl, 8 mM Na_2_HPO_4_, 1.5 mM KH_2_PO_4_) containing 1 µM free biotin. The phage library (∼1×10^10^ phage particles) was first added to the wells coated with NeutrAvidin™ to subtract out non-specific or NeutrAvidin™ binders from the pool during a 1 h incubation at room temperature (RT). The subtracted phage library was incubated with the blocked target for 1 h, followed by six washes with PBS+0.1% Tween 20 (PBST) and six washes with PBS. Phage particles bound to the target were eluted using 100 mM glycine-HCl (200 µL; pH 2.0), neutralized with 2 M Tris-base (12 µL; pH 10.0) and used to infect 800 µL of TG1 *Escherichia coli* cells (Agilent Technologies, Santa Clara, CA) at mid-log phase [optical density (OD) at 600 nm = 0.5] for 40 min at 37°C. The cells were plated after infection on Petri plates containing Luria Broth (LB; 10 g/L tryptone, 5 g/L yeast extract, 10 g/L NaCl) and 1.5% agar. After overnight incubation at 37°C, the colonies were scraped off, and ∼5×10^8^ cells were transferred into 50 mL of LB media containing 50 µg/mL carbenicillin, and grown for 2–3 h at 37°C, with 250 rpm shaking. When the culture reached mid- log phase, the cells were infected with helper phage (M13K07 or K07, depending on the library used for selection; New England BioLabs, Ipswich, MA) at a multiplicity of infection (MOI) of 20 for 1 h, transferred to fresh LB media +50 µg/mL carbenicillin and 50 µg/mL kanamycin, and incubated overnight at 30°C. The next day, phage particles were harvested from the culture supernatant, precipitated with 5% PEG 8000 and 300 mM NaCl (final concentrations), dissolved in PBS, and used for the next round of affinity selection. The second and third rounds of affinity selection were conducted in the same manner, except that only 50 µL of biotinylated peptides (10 µg/mL) were used to coat the wells, only 1/2 the volume of the eluted phage was used to infect bacterial cells after round #2, and only 1/4 of the volume was used in round #3. After the third round of affinity selection, 96 clones were propagated as phage and analyzed by an enzyme-linked immunosorbent assay (ELISA), thereby identifying clones that display scFvs that bind the peptide target. Positive binding clones were sequenced, and specificity tests were performed.

### 2.4. Cloning into expression vectors

#### 2.4.1. Subcloning into yeast expression vector

In both phage-display libraries, the scFv coding regions are bounded by *Hind* III and *Sal* I [52] or *Nco* I and *Not* I [53] restriction endonuclease sites. To clone these coding regions into pPICZαC′ expression vector, the phagemid vectors were digested with their respective restriction enzymes (New England BioLabs), the released scFv DNA fragment was isolated (Qiagen, Valencia, CA), and ligated with the similarly digested vector DNA using 1 unit of T4 DNA ligase (New England BioLabs) for every 1 µg of digested vector. The resulting recombinant DNA was used to transform electrocompetent TG1 cells (Lucigen, Middleton, WI) and plated on low-salt LB (1% tryptone, 0.5% yeast extract, 0.5% NaCl, pH 7.5)+25 µg/mL Zeocin™ (Invitrogen, Carlsbad, CA)+15 g/L agar plates and grown overnight at 37°C. The positive clones were digested with *Pme* I (New England BioLabs) to linearize the DNA and used to transform the X33 strain of *P. pastoris*, which was grown according to the manufacturer's instructions (Invitrogen). The transformants were grown with varying concentrations of Zeocin™ (0.1 mg/L and 1 mg/L) in YPD medium [Yeast extract Peptone Dextrose medium: 1% yeast extract, 2% peptone, 2% dextrose (Sigma, St. Louis, MO)] to select for clones that carried multiple integration events.

#### 2.4.2. Subcloning into mammalian expression vector

The scFv A10 was PCR amplified from the pPICZαC′ vector using cloning primers described above, thereby introducing an *Nco* I/*Not* I site on the 5′ and 3′ ends, respectively. The resulting PCR product was digested with the above restriction enzymes and ligated into the similarly digested pBIOCAM5 vector, used to transform TG1 cells, and recombinants were sequenced. The plasmid DNAs from positive recombinant clones were purified using Pure Link™ HiPure Plasmid Filter Maxiprep Kit (Invitrogen), extracted with phenol-chloroform, and precipitated with ethanol. Approximately 20 µg of filter-sterilized DNA was added to 4 mL of Freestyle media (Invitrogen, #12338-018) and 40 µL of PEI (polyethylenimine, Polyscience Inc., #23966), vortexed, and incubated at RT for 10 min. The mixture was added to 50 ml of FreeStyle™ 293F (variant of HEK 293 cells, Invitrogent #R790-07) cells (1×10^6^ cells/ml). Transiently transfected cells were grown at 37°C, 10% CO_2_ on an orbital shaker platform rotating at 135 revolutions per min (rpm) for 7 days before harvesting of the secreted protein.

#### 2.4.3. Subcloning into pAT224 vector

To construct fusions of the peptides to maltose binding protein (MBP), the α1 and β2 peptide sequences were designed as two sets of overlapping primers that would insert the respective peptide sequences at the *C*-terminus of the MBP gene in pAT224 (gift of Dr. Andreas Plückthun). This protocol is described elsewhere [51].

### 2.5. Protein and phage ELISA

To amplify the phage particles displaying the recombinant scFv variants, TG1 bacterial cells (5 mL) harboring the phagemid DNA were infected at mid-log phase with M13K07 helper phage, at a MOI of 20, and grown for 1 h at 37°C, with 150 rpm shaking. Infected cells were centrifuged, the pellet was resuspended in fresh LB medium that contained 50 µg/mL carbenicillin and 50 µg/mL kanamycin, and phages were amplified overnight at 30°C in a shaking incubator (250 rpm). All of the phage ELISA steps were performed at RT. Biotinylated peptides (50 µL, 10 µg/mL) were immobilized on Nunc MaxiSorp flat-bottom 96 well plates via NeutrAvidin™ Biotin binding protein (50 µL, 10 µg/mL) and blocked with 2% skim milk in 1× PBS with 1 nM free biotin (200 µL per well). After washing with PBS, the wells were incubated with the phage supernatant (50 µL) or protein scFv A10 (6 µM) or scFv-Fc A10 (1 µM) for 1 h, and washed three times with PBST. The binding phage particles were detected using anti-M13 antibody conjugated to horseradish peroxidase (HRP) (GE Healthcare, Piscataway, NJ) while binding protein molecules were detected by anti-Flag antibody conjugated to HRP (GE Healthcare, Piscataway, NJ) diluted 1∶5000 in PBST. After washing away of the non-bound antibody molecules, the chromogenic substrate for HRP, 2,2′-Azino-bis (3-ethylbenzothiazoline-6-sulfonic acid) diammonium salt (ABTS, Thermo Fisher Scientific) supplemented with hydrogen peroxide was added (45 µL per well), and the absorbance at 405 nm was measured on a POLARstar OPTIMA microtiter plate reader (BMG Labtech, Ortenberg, Germany).

### 2.6. Protein Expression

#### 2.6.1. Protein expression in Pichia pastoris

For production of scFv protein, *P. pastoris* X33 cells, harboring the multicopy integrants, were grown overnight at 30°C in 50 mL BMGY (Buffered Glycerol-complex medium; 1% yeast extract, 2% peptone, 100 mM potassium phosphate, pH 6.0, and 1.34% YNB (Yeast Nitrogen Base, Invitrogen), 4×10^−5^% biotin and 1% glycerol) at 300 rpm. The next day, the cells were pelleted (2500 rpm, 10 min, RT), resuspended in 200 mL BMMY (Buffered Methanol-complex medium: 1% yeast extract, 2% peptone, 100 mM potassium phosphate, pH 6.0, 1.34% YNB, 4×10^−5^% biotin and 0.5% methanol), and grown for three days at 300 rpm; supplementing methanol (25 mL; 0.5%) was added each day to compensate for loss by evaporation. The culture medium was collected after the third day by centrifugation of cultures (2500 rpm, 10 min, RT), and the secreted scFvs were isolated by immobilized metal affinity chromatography, using Ni-NTA conjugated agarose beads (Qiagen) and elution with PBS containing 200 mM imidazole (Sigma). The purified proteins were diluted 1∶50 in PBS, tested by ELISA as described above, and detected using HRP conjugated anti-Flag antibody (diluted 1∶10,000 in PBS; Sigma).

#### 2.6.2. Protein expression in FreeStyle™ 293F cells

Transiently transfected FreeStyle™ 293F cells were grown for 7 days as described above. The culture supernatant was collected by centrifugation (4000 rpm, 5 min, 4°C). To the supernatant was added binding buffer (0.05 M sodium phosphate monobasic, 0.05 M sodium phosphate dibasic, 0.15 M sodium chloride and 10 mM imidazole), and 1× Protease Inhibitor Cocktail (Roche, Indianapolis, IN, #14424700). The resulting mixture was added to 1 ml His60 Ni Superflow™ Resin (Clontech Laboratories, Inc., #635662) and tumbled overnight at 4°C. The beads were washed three times with wash buffer (100 mM sodium chloride, 10 mM imidazole) and the scFv-Fc proteins eluted with 200 mM sodium chloride and 400 mM imidazole (pH 8).

#### 2.6.3. Expression of MBP-fusion proteins

One hundred mL of 2×YT/CB media was seeded with 1 mL of the overnight starter culture and grown to mid-log phase (OD_600 nm_ = 0.4–0.5), when 50 µL of 1 M isopropyl β-D-1-thiogalactopyranoside (IPTG) was added to the culture to yield a final concentration of 0.5 mM IPTG. The culture was incubated at 30°C, with shaking (250 rpm), overnight. The culture was then centrifuged at 10,000 rpm at 4°C for 10-min, and the cell pellet was harvested and stored at −20°C. The frozen cell pellet was incubated with 5 mL of BugBuster MasterMix [per 10 mL: 1 mL BugBuster (Novagen, San Diego, CA; #70921), 5 µL 2000× Benzonase (Novagen, #D00138360), 200 µL 50× protease inhibitor cocktail (Roche) and 2 µL 5000× lysozyme (Novagen, #71110-3)] for every 1 g of cell pellet in 37°C for 10 min. The tube containing the mixture was then placed on ice and sonicated with a Sonic Dismemberator (Fisher Scientific, Model 500; tip from Branson Inc.) at 50% intensity with 30-sec intervals for a total of 20 min. The cell lysate was then centrifuged at 15,000 g for 10 min; the supernatant was supplemented with 3 mL of His60 Ni Superflow™ Resin (Clontech), and rocked at RT for 1 h. The beads were washed thrice, each time with 50 mL of each of three Wash Buffers (WB1, WB2 and WB3, respectively). Buffer WB1 consisted of 0.3 M sodium chloride, 50 mM sodium phosphate monobasic, 0.5% Tween-20 and 10 mM imidazole; WB2 and WB3 were identical to WB1 except for the concentration of imidazole (15 mM in WB2, and 20 mM in WB3). Bound MBP-fusion protein was eluted with 5 mL of elution buffer (250 mM imidazole, 0.3 M sodium chloride, 50 mM sodium phosphate monobasic).

### 2.7. SRU Biosystem's BIND® READER

Streptavidin-coated 96-well BIND® plate-based biosensors (SRU Biosystems, Woburn, MA) [50] were used to estimate dissociation constants of isolated scFvs, according to the manufacturer's protocol. PBS (50 µL/well) was added to the label-free plate coated with SA, and the plate was read in the plate-based reader, BIND®SCREENER (SRU Biosystems) until wavelength fluctuations were less than 5 picometer/min. Biotinylated (50 µL at 10 µg/mL) peptides were then added to wells and incubated at RT for 1 h. Plates were read for 5 min (or until the signal reached a plateau) and then blocked with 50 µL of PBST+5% BSA. After blocking, 50 µL of elution buffer was added and the plate read as before, at which time the reader was paused and 50 µL of different concentrations (A10: 0.03–17.5 µM; A7: 0.015–17.5 µM; G8: 0.03–17.5 µM and G11: 0.008–15 µM) of scFvs were added to the wells; the reading then resumed for another 5 min. After a 1 h incubation at RT, the plate was read again until the reaction reached saturation (i.e., until the signal reached a plateau). The change in peak wavelength value (before and after 1 hr incubation with scFvs, compared to the background-subtracted data) was plotted against concentrations, and the dissociation constants were calculated using SRU's EMS software [57].

### 2.8. Fluorescence Polarization (FP)

For FP, 6 µM of FITC-labeled α1 peptide and 4 µM of FITC-labeled β2 peptide were incubated with varying concentrations of A10 (0–35 µM), A7 (0–35 µM), G8 (0–35 µM) or G11 (0–10 µM) scFvs and A10 scFv-Fc (0–1 µM), respectively, in elution buffer (125 µL; PBS+200 mM imidazole) overnight at 4°C or 2 h at RT. The resulting complexes were transferred to Corning® 96 well half-area, polystyrene black plates (Corning, NY), and FP values were measured using the POLARstar OPTIMA plate reader from BMG LABTECH (BMG LABTECH Inc., Cary, NC). The resulting millipolarization (mP) values were plotted against concentration and dissociation constants calculated using OriginPro8.5 software (OriginLab Corp., Northampton, MA).

### 2.9. Immunofluorescence labeling and electrophysiology

#### 2.9.1. Xenopus laevis oocytes expressing human α1β2γ2 GABA_A_ receptors (rat α1, rat β2 and human γ2 subunit sequences) were prepared as described previously [58], [59], [60]

Briefly, mRNA for each subunit, prepared by *in vitro* transcription (mMessage mMachine kit; Ambion Inc., Austin, TX) of linearized plasmids, was injected into the oocytes with a Nanoject II microinjector (Drummond Scientific Co., Broomall, PA). Oocytes were analyzed for GABA receptor expression after 24–72 h incubation at 16°C in physiological saline (100 mM NaCl, 2 mM KCl, 2 mM CaCl_2_, 1 mM MgCl_2_, 5 mM HEPES, and 10 mM glucose, pH 7.2–7.4) containing 0.1 mg/ml gentamycin.

Immunofluorescence labeling of oocytes, either expressing α1β2γ2 GABA_A_ receptors, or non-expressing negative controls, involved a blocking step with 10% fetal bovine serum diluted in physiological saline (1 h, room temperature). The blocked oocytes were then incubated overnight at 16°C with control primary antibody (anti-α1: Abcam, Cambridge, MA); scFvs (A10: 400 nM, G8 and A7: 1 µM, G11: 300 nM), with scFvs that had been pre-absorbed with 0.1 mg/mL of cognate peptide (pre-absorption: 4°C, 16 h, with agitation), or without primary antibody. The secondary antibody was monoclonal mouse M2 anti-Flag, FITC-conjugated, 1/1000 (Sigma), and was used with a 1 h incubation at RT. Images were obtained on a Leica DM-IRE2 confocal microscope at 20× magnification.

Electrophysiology of oocytes was performed by recording membrane current responses to the presentation of 10 µM GABA using a two-electrode voltage-clamp apparatus (GeneClamp 500B amplifier; Axon Instruments, Foster City, CA, USA) and Clampex 8.2 software (Axon Instruments). Recording procedures were similar to those previously described [42], [60]. Prior to electrophysiological testing, oocytes were subjected to conditions similar to those used for immunofluorescence (i.e., untreated; incubated with scFv or control primary antibody only; or incubated with scFv followed by secondary antibody).

#### 2.9.2. Mouse retina

Retinal tissue was obtained from a C57BL/6J mouse (Jackson Laboratories, Bar Harbor, ME), and fixed in 4% paraformaldehyde, cryopreserved in sucrose, then sliced in Optimal Cutting Temperature medium (OCT, Tissue-Tek). The thickness of the retinal cryosections was 16 µm. scFvs, which had been biotinylated *in vitro* using EZ-Link Sulfo-NHS-LC-LC-Biotin (Thermo Scientific), were used for immunofluorescence staining of cryosections. Detection of immunocomplexes was performed using Cy5-conjugated streptavidin, 1/200 (Invitrogen) or Cy5 conjugated Goat Anti-Rabbit IgG (for positive control; Abcam). Slides were mounted using Vectashield H-1000 medium (Vector Laboratories, Burlingame, CA), and images (20× magnification) obtained by confocal microscopy (Leica DM-IRE2).

### 2.10. Dot Blot

Immobilon-FL polyvinylidene fluoride (PVDF) membranes (Millipore) were pre-activated by immersing in 100% methanol for 30 sec and then washing in dH_2_O for 2 min. The membrane, along with two 0.5 mm filter pads, was soaked in PBS for 10 min in order to equilibrate in the buffer. A dry filter pad was placed on top of a bench, followed by the soaked filter pad and finally the PVDF membrane. Various amounts (1 µg, 0.5 µg, 0.25 µg, 0.125 µg, 0.0625 µg, 0.03 µg, 0.015 µg, 0.0075 µg) of MBP-fusion proteins were then adsorbed onto their respective grids in the membrane and left to dry on the bench, at RT, for 2 h. The blot was then blocked in 15 mL of 5% non-fat dry milk in PBS for 1 h in RT, followed by three washes in PBS for 5 min. Approximately 10 nM each of A10 scFv, A10 scFv-Fc and anti-α1 antibody (Abcam) was added to the appropriate strips and incubated overnight at 4°C with shaking. The binding of scFv and scFv-Fc versions of A10 was detected with α-Flag-800CW (1∶15,000; Rockland Immunochemicals, Gilbertsville, PA) and anti-α1 with goat, anti-rabbit HRP (1∶5,000; Pierce) for 45 min at RT. The membrane was washed three times and imaged with the Li-COR imager dual channel (800 nm and chemiluminescent) device (Odyssey Li-COR Biosciences; Lincoln, NE).

## Results

### 3.1. Isolation of anti-GABA_A_ receptor specific scFvs

**Figure 1:**
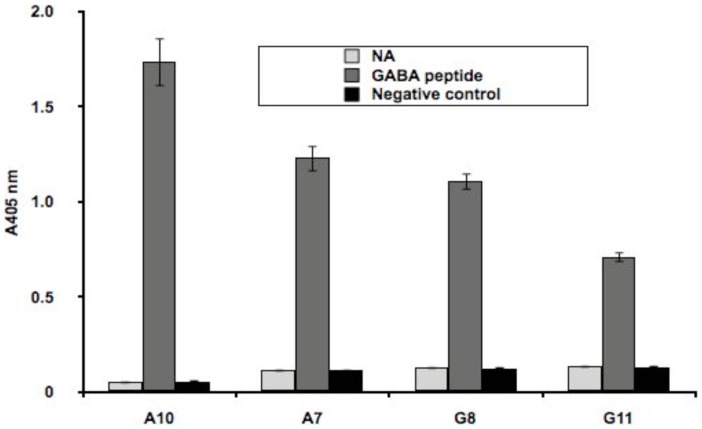
Binding of phage-expressed scFvs affinity selected with GABA subunit peptides. Equal amounts of biotinylated target peptides or non-target peptide (negative control) were captured on NeutrAvidin™ (NA) coated microtiter plate wells, and after washing the binding of equivalent amounts of phage particles, displaying different scFvs, was monitored by ELISA. A biotinylated anti-Flag antibody was used to normalize the amounts of scFv-displaying phage particles added to each well. Error bars correspond to the standard deviation of triplicate measurements of the optical density of the wells at 405 nm wavelength. A7, G8 and G11 are anti-β2 binders while A10 is the anti-α1 binder.

To obtain scFvs that bind specifically to the GABA_A_ receptor subunits, we performed affinity selection experiments with libraries [52], [56] that displayed naïve scFv on the surface of bacteriophage M13 particles. We used biotinylated peptides (15- and 19-mers, respectively) corresponding to the *N*-terminal extracellular domains of α1 and β2 GABA_A_ receptor subunits (residues 1–15 of the rat α1 subunit, and residues 1–19 of the rat/human β2 subunit) as targets for affinity selection along with biotinylated peptides from other retinal membrane proteins, GBB5 and Pep1 (section 2.2). Briefly, three rounds of affinity selection were performed on NeutrAvidin™/streptavidin-coated Nunc polystyrene plates, which had captured either biotinylated peptide or negative control peptides. The strongest binders were demonstrated in an ELISA to bind specifically to their cognate peptides (Fig. 1), and their primary structures are shown in Figure S1.

### 3.2. Expression of anti-GABA_A_ receptor-specific scFvs in Pichia pastoris

The binding scFvs isolated from a phage-display library were subcloned into a *P. pastoris* expression vector for soluble expression into the culture medium. After three days of induction with methanol, the culture medium was recovered, and the secreted scFvs were purified using Ni-NTA coated agarose beads, as described in section 2.5. The purified proteins were resolved in a 12% SDS-PAGE gel and determined to be >98% pure (Figure S2A). Protein expression yields (Table S1) ranged from 4 to 22 mg/L of flask culture. The soluble proteins were confirmed by ELISA to have the same binding properties as their phage-displayed variants (compare Fig. 1 and Figure S2B).

### 3.3. Characterization of binding strengths of anti-GABA_A_ receptor scFvs

**Figure 2:**
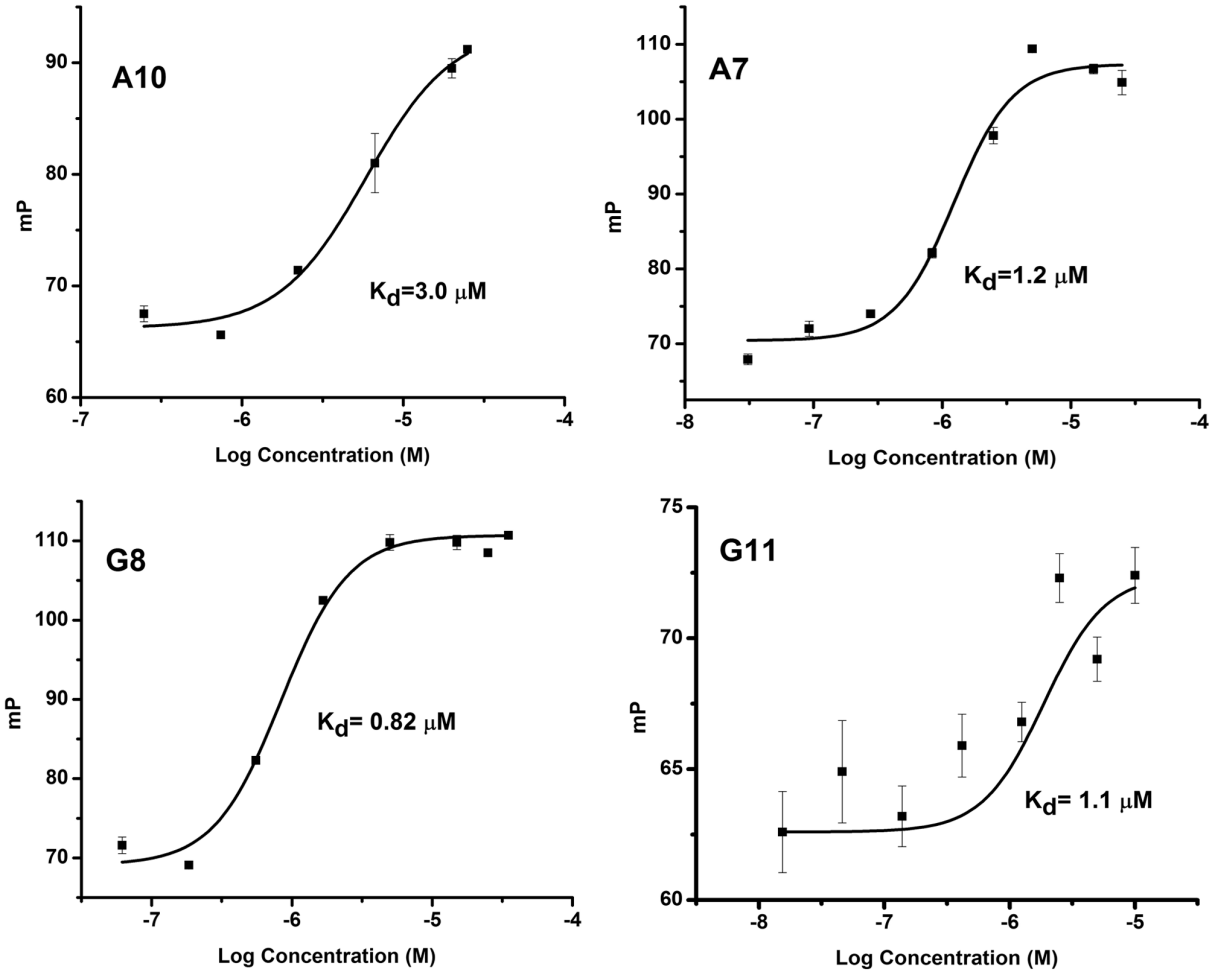
Binding affinities of anti-α1 and β2 scFvs measured using fluorescence polarization. Purified scFv proteins were incubated with 6 or 4 µM (α1 or β2, respectively), of FITC-conjugated target peptide. Changes in polarization were measured in millipolarization (mP) units and plotted against concentration of the scFv. The K_d_ value of one representative trial is shown. Error bars correspond to standard deviations for duplicate measurements.

To characterize the affinities of the isolated scFv binders for their cognate peptide, we determined the dissociation constants (K_d_) of the scFvs by both FP and with the SRU BIND® Reader. For the FP measurements, we followed the change in polarization of the FITC-labeled peptide over a range of concentrations of each scFv. The increase in (mP) units corresponding to increasing scFv concentrations (in µM) was plotted as a “dose-response curve” using OriginPro8.5 software (Fig. 2). The K_d_ values determined by FP were corroborated with the BIND® Reader (Table 1). As indicated by Table 1, K_d_ values determined by the two methods correlate well, and the K_d_ value for the anti-α1 binder is about 3-fold higher than those for the anti-β2 scFvs (∼3 µM for A10 versus ∼1 µM for A7/G8/G11). In addition, all binders have K_d_s in the low micromolar range (1–3 µM), which is in the expected range of K_d_s for scFvs from naïve libraries (10^−5^ to 10^−8^ M) [61], [62].

**Table 1:** Binding affinities of anti-GABA_A_ receptor subunit scFvs.

scFv	FP (µM)	BIND (µM)
	K_d_ ± SD	K_d_ ± SD
A10	3.5±0.5	2.4±1.3
A7	1.1±0.1	1.4±0.8
G8	0.97±0.1	1.6±0.5
G11	1.4±0.3	0.86±0.6
A10-Fc	0.026±0.0079	n.d.

Dissociation constants (µM) as measured by BIND® Reader or FP are shown. First four rows show dissociation constants of scFvs and the last one that of the scFv-Fc reagent. SD, standard deviation of three (BIND) or two (FP) independent trials (except for A10-Fc; 3 independent trials); n.d., not determined.

### 3.4. Effect of scFv binding on channel activity of GABA_A_ expressing oocytes

**Figure 3:**
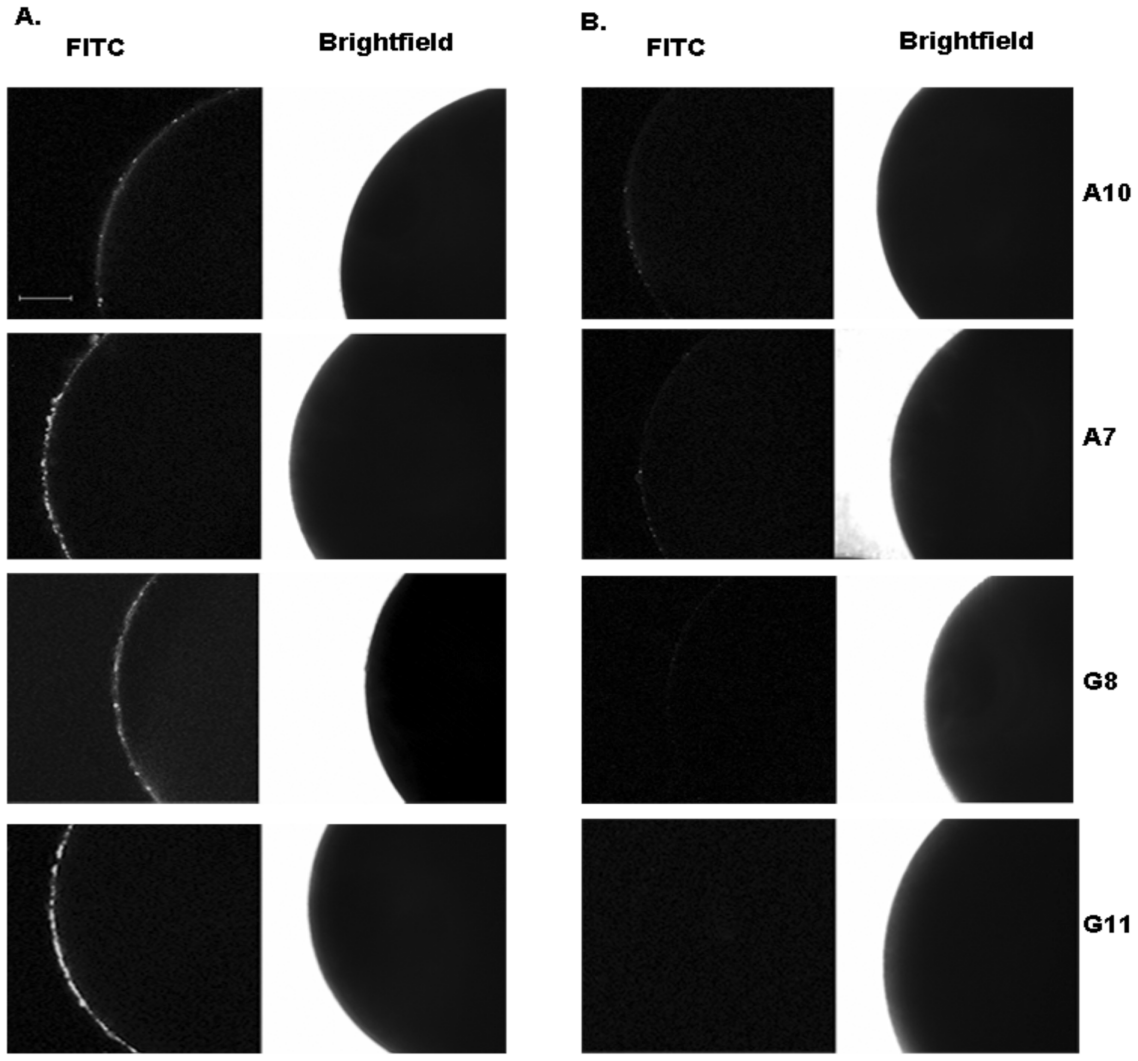
Immunofluorescence staining of GABA_A_ receptors expressed in *Xenopus laevis* oocytes. *X. laevis* oocytes were injected with 50 nL of GABA_A_ receptor mRNA (A) or buffer (B), and later incubated with 70 nM anti-α1 (A10) and 100 nM β2 (A7, G8, G11) scFvs, and binding of the scFvs to the cell surfaces was detected with a mouse anti-Flag antibody conjugated to FITC. Representative images are shown for each condition; four independent replicates were performed for each condition. Scale bar depicts 150 µm.

To investigate binding of the isolated peptide-binding scFvs to intact GABA_A_ receptors, we performed immunofluorescence on GABA_A_ expressing and control (non-expressing) *Xenopus* oocytes. GABA_A_-expressing and (as controls) non-expressing oocytes were analyzed under four experimental conditions: (1) following treatment with a given scFv; (2) treatment with the scFv pre-incubated with its cognate peptide; (3) treatment with the scFv and secondary antibody; and (4) treatment with the scFv pre-incubated with cognate peptide and secondary antibody. For all of the investigated scFvs, incubation of anti-GABA_A_ scFvs with the GABA_A_ expressing and non-expressing oocytes, followed by visualization with FITC-conjugated secondary antibody, yielded a fluorescence signal at the surface membrane specifically of the GABA_A_ expressing oocytes (Fig. 3). The faint halo seen in the non-expressing oocytes treated with scFvs is most probably due to non-zero background fluorescence, as it is far weaker than the positive signal seen for GABA_A_-expressing oocytes treated with scFvs (compare Fig. 3A to 3B). This positive signal was absent when the receptor-expressing oocytes were incubated with scFvs that had been pre-incubated with their cognate peptide (Figure S3), indicating the dependence of the signal on receptor recognition by the scFvs. Therefore, the peptide-specific scFvs isolated in this study are capable of binding intact GABA_A_ receptor.

To determine whether the binding of a given scFv to GABA_A_ receptors expressed on the oocyte surface affects receptor activity, we employed oocytes that had been treated as described in the immunofluorescence experiments, and examined membrane currents elicited by GABA at a concentration (10 µM) that nominally produces a response of peak amplitude well below response saturation [63], [64], [65]. The incubation periods required for the treatments with scFvs (see section 2.9 for details) precluded direct comparison of the effects of these treatments on a single oocyte. Accordingly, we determined the GABA-elicited peak amplitude and time course of responses recorded from GABA_A_ expressing oocytes subjected to a given treatment, and compared these response properties across different treatments. In the first of these comparisons, we determined average peak amplitudes of the 10 µM GABA response of oocytes treated with a given scFv (Rmax, treated), and normalized these values to the average peak amplitudes obtained from control GABA_A_ expressing oocytes (i.e., oocytes incubated in unsupplemented physiological saline, studied on the same experiment day; Rmax, control). Entries in columns 1 and 2 of Table 2 show the normalized peak amplitude of the GABA-elicited response for a given treatment. Entries in columns 3 and 4 indicate normalized peak amplitudes of the GABA-elicited response for each scFv after incubation with secondary antibody. This was done to study the effect on channel dynamics of adding a macromolecular, sterically bulky moiety to the scFv. The results indicate that the membrane current responses of oocytes incubated with and without the secondary antibody reagent are not substantially different (compare normalized peak amplitude entries of column 3 and 4 of Table 2).

**Table 2:** Potentiation factor and goodness-of-fit (Λ) of respective waveforms of GABA_A_ receptors expressing oocytes.

		(1) scFv alone	(2) scFv+Peptide	(3) scFv+Sec	(4) scFv+Peptide+Sec
A7	Normalized Peak Amplitude	0.513±0.076	0.358±0.148	0.655±0.033	1.304±0.213
	Λ Value	0.99±0.00	0.89±0.03	0.93±0.13	0.82±0.09
A10	Normalized Peak Amplitude	0.857±0.765	0.757 (Single oocyte data)	1.564±0.866	1.223±0.517
	Λ Value	0.85±0.19	0.81 (Single oocyte data)	0.88±0.00	0.86±0.02
G8	Normalized Peak Amplitude	1.346±1.115	1.419±0.937	0.566±0.228	1.259±0.711
	Λ Value	0.86±0.01	0.89±0.00	0.89±0.01	0.89±0.10
G11	Normalized Peak Amplitude	0.698±0.623	0.718±0.415	0.740±0.546	0.528±0.280
	Λ Value	0.97±0.00	0.91±0.03	0.94±0.01	0.94±0.01

Membrane current responses of GABA_A_-expressing oocytes to 10 µM GABA are depicted as normalized peak amplitudes and as goodness-of-fit (Λ values). Errors shown are standard deviations of 3 independent trials. Entries are based on results obtained from 3–8 oocytes, except for A10 entries in column 2 of the table (one oocyte).

The second measure of comparison performed was to analyze the effects of a given treatment on the waveform (i.e., kinetics) of the GABA-elicited response. For this analysis, we first determined the value of the parameter α, a scaling factor, defined as the inverse of the normalized peak amplitude described above [(Rmax, treated)/(Rmax, untreated)]. Using α determined for a given treatment, we compared the waveform of the treated oocyte with that of the relevant control response using a relation similar to that conventionally used to test the goodness-of-fit of an analytical curve with a family of experimentally determined data points (equation 1)
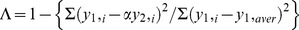
(1)where, y_1,i_ is the amplitude of the response (y_1_) of the untreated oocyte at a given time t_i_; y_2,i_ is the amplitude of the response (y_2_) of the treated oocyte at the same time t_i_; the expression (y_1,i_ - αy_2,i_) affords a match of the peak amplitudes (occurring in general at different times) of the responses y_1_ and y_2_; y_1_,_aver_ is the average amplitude of the response from the untreated oocyte; the summations are over all times t_i_; and the parameter Λ is a measure of the kinetic correspondence of the two waveforms (Table 2).

The tabular data indicate differing normalized peak amplitudes among GABA_A_-expressing oocytes treated with the different scFvs. However, for all of these investigated oocytes (both scFv-treated and untreated), the peak amplitude of the GABA-elicited response was 60 nA or greater (data not shown). As a control, for each of the conditions of scFv treatment described in Table 2, we similarly tested at least one non-expressing oocyte. In all cases the non-expressing oocyte exhibited no detectable response (i.e., <3 nA) to the presentation of 10 µM GABA (data not shown). The large standard deviations among the data for normalized peak amplitudes likely reflected, at least in part, differences in GABA_A_ receptor expression. Determinations of Λ provide further information on the effect of scFv treatment. They show, specifically, that point-by-point comparison of peak-matched waveforms of untreated and treated oocytes yield goodness-of-fit values near unity, indicating high similarity in waveform kinetics, consistent with minimal or no effect on receptor channel activity upon treatment with scFvs. While physiological control of the receptor by a combinatorially-derived affinity reagent has been observed in previous studies [66], the absence of an effect of the scFvs studied here on the GABA_A_ electrophysiological activity is consistent with the suitability of these scFvs as the anchor component of a prospective neuromodulator (see Introduction).

### 3.5. Recognition of native receptor expressed in the mouse retina

**Figure 4:**
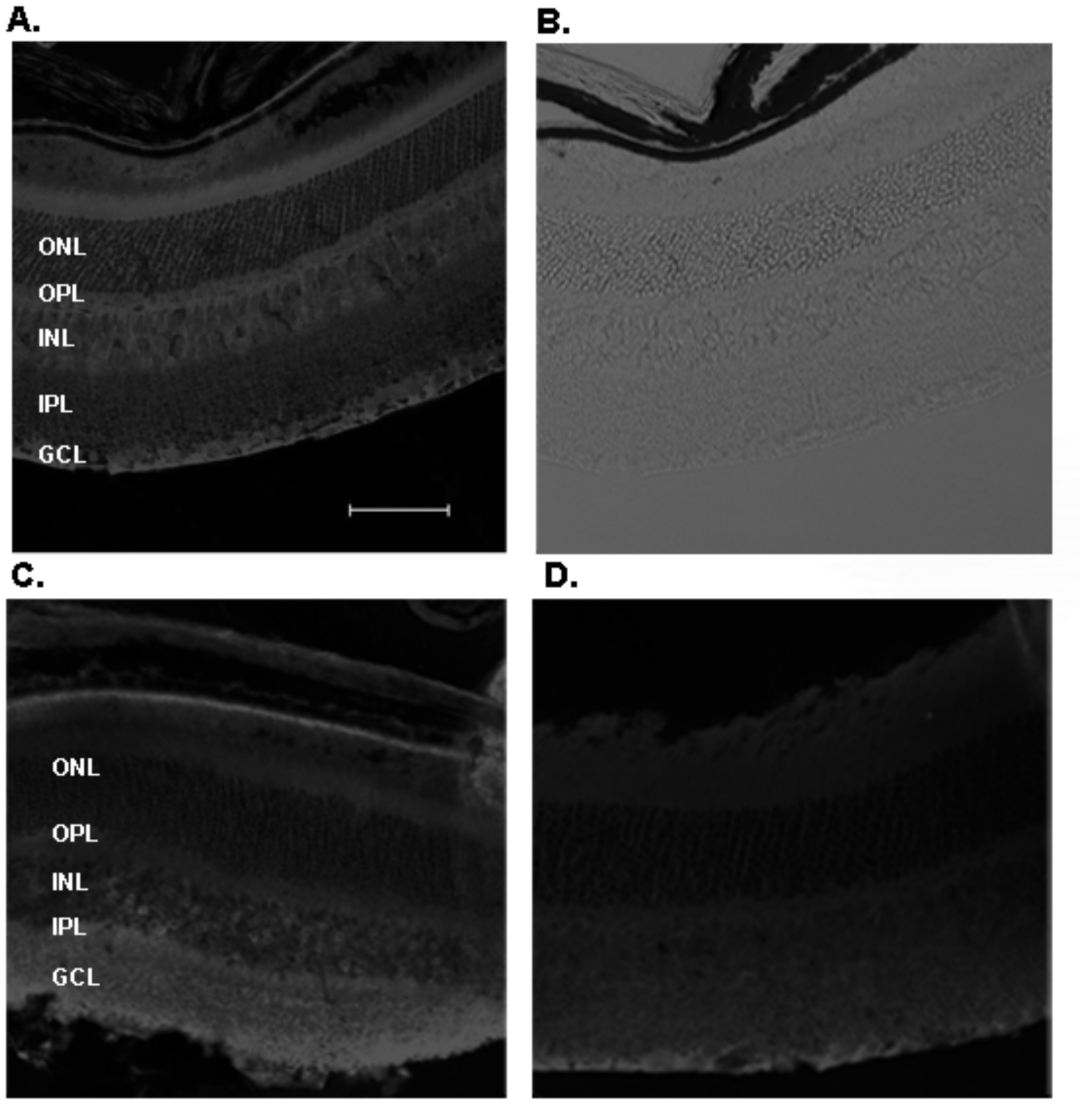
Binding of scFvs to native GABA receptors in mouse retinal sections. Immunohistochemistry of cryosections of mouse retina incubated with 600-α1 A10 scFv, (A and B), 300 nM biotinylated ZF130H1, negative control scFv (D) or 47 nM of anti-GABA_A_ positive control antibody (C). Streptavidin (SA)-Cy5 or anti-rabbit IgG-Cy5 was used as the secondary detection reagent. Panels A, C and D are images obtained under fluorescent light (Cy5 filter), while panel B shows the image acquired under bright light. Representative images from three independent trials are shown. Scale bar depicts 80 µm. Legend: GCL; Ganglion cell layer, INL; Inner nuclear layer, IPL; inner plexiform layer, ONL; outer nuclear layer, OPL; outer plexiform layer.

From the above results, it is clear that the scFvs generated against synthetic peptides were capable of recognizing intact receptors in a heterologous system. The next step in characterizing these scFvs, was to test if they can bind native receptors in the retina. Immunohistochemistry and *in situ* hybridization studies using subunit specific monoclonal antibodies and oligonucleotides, respectively, have shown that GABA_A_ receptors containing the α1 subunit are expressed in retinal amacrine cells and bipolar cells [8], [10], [11], [12] (reviewed in [67]). In immunohistochemistry experiments on cryosections of mouse retina, we investigated the binding of A10, the α1-specific scFv described above. The results, shown in Figure 4A, indicate distinct staining of the ganglion cell and inner plexiform layers (bright regions in the image), consistent with previously reported data [9], [13], [68]. A similar staining pattern was obtained with commercially available anti-α1 monoclonal antibody (Fig. 4C). The small differences in staining patterns obtained with the present scFv and the commercial antibody can be explained by possible differences in the epitopes used to generate the two antibodies. Further evidence that the staining by A10 is due to specific recognition of the receptor came from an experiment in which we tested an scFv raised against an unrelated target [ZF130H1; Suppression of tumorigenicity13 (Hsp70 interacting protein of zebrafish) [69]]. This ZF130H1-directed scFv did not yield a layer-specific staining pattern in retina (Fig. 4D). These data demonstrate that the scFv A10 recognizes GABA_A_ α1 subunit in the mouse retina.

### 3.5. Increasing sensitivity of scFvs in western blots by dimerization

**Figure 5:**
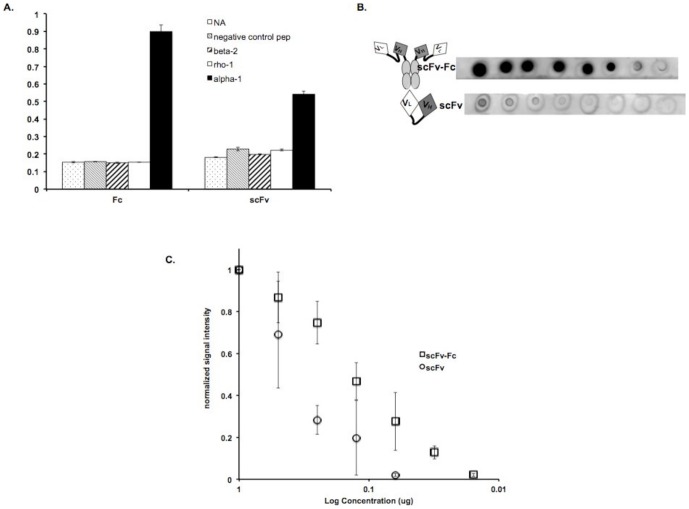
Comparison of detection limits of scFv and scFv-Fc. A. Binding specificity of equimolar concentrations of scFv-Fc and scFv A10, to different biotinylated peptides was detected in an ELISA using anti-Flag antibody conjugated to HRP. Signal intensities were measured at 405 nm and error bars indicate standard deviation of duplicate trials. NA, NeutrAvidin™; negative control pep, retinal protein Pep 1 peptide; beta 2, GABA_A_ receptor subunit β2 peptide; rho 1, GABA_c_ receptor subunit ρ pep tide and alpha 1, GABA_A_ receptor subunit α1 peptide. B. Dot blots of varying concentrations of MBP-α1 proteins treated with 10 nM A10 scFv-Fc (top row) and scFv (bottom row). Circles around signal spots indicate pencil markings of protein addition sites. C. Signal intensities normalized to highest signal obtained for each trail are plotted against log of concentration for scFv-Fc and scFv. Error bars depict standard deviations. n = 3.

The above results indicate that the generated anti-peptide scFvs were capable of recognizing the native receptor in cell and tissue staining experiments. However, these reagents did not exhibit robust recognition of denatured receptor epitopes in western blots (data not shown), presumably as a consequence of their high (micromolar) K_d_ values. In order to improve the binding affinity of the isolated scFvs, we generated dimeric versions by subcloning the anti-α1 scFv, A10 in the pBIOCAM5 vector as described in section 2.3.2. The resulting dimeric scFv-Fc reagent was expressed and purified from FreeStyle™ 293F cells (section 2.5.2). The dimer retained the subunit specificity of its monomeric units as measured by ELISA (Figure 5 a). The anti-α1 scFv-Fc A10, exhibited a K_d_ of 26±7.9 nM (Table 1), which is an ∼135-fold increase in apparent affinity over the scFv variant (Table 1). In order to test the sensitivity of this dimeric scFv-Fc in dot blots, varying concentrations of the MBP-α1 peptide fusion protein were blotted onto nitrocellulose membranes and treated with 10 nM of scFv, scFv-Fc or commercially available anti-α1 antibody as described in section 2.8. The results (Figure 5 b and c) show that the scFv-Fc version has greater sensitivity than the scFv version. Sensitivity of this dimeric scFv is very close to that achievable by the commercially available IgG (data not shown). Moreover, Fig. 5c indicates that the increase in sensitivity over its scFv counterpart is ∼10-fold (see X-intercepts of both sets of data).

## Discussion

The present study demonstrates the successful isolation of scFvs against GABA_A_ receptor subunits by phage display technology using synthetic peptides. These scFvs bind to their cognate peptide specifically, with minimal or no cross-reactivity to peptides of other GABA subunits. Significantly, these peptide-raised scFvs specifically bind intact GABA_A_ receptors expressed in *Xenopus* oocytes, and appear not to substantially alter the receptor's electrophysiological activity. Furthermore, A10, the α1-specific scFv, labels mouse retina in layers expected to contain GABA_A_ receptors. The results additionally show that dimerization of the A10 scFv yields a more robust reagent, as evidenced by the10-fold enhancement of sensitivity in dot blots (Fig. 5b) and the more than 100-fold reduction in K_d_ value (Table 1)

The immediate motivation of the present study was development of an scFv anchoring component for a GABA_A_-specific modulating device [42] (also see [70]). Beyond this potential application, the reagents developed here have multiple possible uses in which the active scFvs can be used as pharmacological modalities. For all of these applications involving receptor targeting, it will be critical to achieve conjugation in which the scFv-targeting moiety preserves the activity of the active ligand. One such method would involve incorporation of a cysteine at a suitable site within the scFv and coupling to a maleimide-terminated receptor ligand (e.g., “MPC100” described in [42]). A distinctly different method, one that avoids potential pitfalls of the cysteine substitution method just noted (e.g. unwanted disulfide bridges), is a chemo-enzymatic method described by Ta et al. [71] utilizing *S. aureus* sortase A enzyme and an LPETG motif incorporated into the anchoring moeity. Here, an LPETG tag is incorporated at the *C*-terminal of the scFv and coupled via sortase A to the active ligand that has been tagged with GGG-amine.

Apart from the above-mentioned applications that employ ligand-coupled scFvs, the uncoupled scFvs might also be used in additional potential applications including the monitoring of expression and trafficking of specific subtypes of GABA_A_ receptor in neuronal tissues, and analysis of the microenvironment of the expressed receptor. Moreover, the present scFvs could prove useful in detecting specific (heteromeric) GABA receptor subtypes, and thus could facilitate identification of different GABA_A_ subtypes that are known to mediate distinct signaling processes in neural tissues.

Although considerable success has been achieved in generating affinity reagents to membrane proteins by performing selections on cells over-expressing these proteins [72] or from selections using purified membrane proteins [73], our method of selecting against synthetic peptides is considerably less tedious, time consuming and requires less optimization steps. Moreover, we have overcome the common issue of poor binding affinity faced by anti-peptide scFvs by a relatively easy (compared to affinity maturation by mutagenic PCR [74]) method of enhancing avidity. This dimerization method can also be exploited to generate bi-specific scFvs, for example, by fusing anti-α1 and anti-β2 scFvs to Fc regions, thereby generating a dimer, which can recognize specific sub-populations of GABA_A_ receptors that contain α1 and β2 subunits.

In conclusion, we, along with other investigators [39], [75] have shown that affinity reagents raised against peptide fragments of membrane proteins are robust binders of the native receptor expressed both heterologously and in animal tissues. We have also shown that the K_d_ values of these binders can be enhanced substantially by dimerization, which enables them to be useful tools in varied pharmacological and biochemical applications. This is significant, as a current bottleneck in studying the structure, function and biology of membrane proteins, specifically channel proteins, is the generation of sufficient quantities of intact and functional proteins [76], which in turn emphasizes the importance of generating robust reagents that can be used to study these proteins [77].

## Supporting Information

Figure S1 **Sequence alignment of the predicted primary structures of anti-GABA_A_ receptor subunit scFvs.** Alignments of the primary structures of the scFv light (L) and heavy (H) chains and their complementarity determining regions, CDR, (boxes) were generated with Clone Manager (SciEd software). Regions of high similarity (>65%) are highlighted in light green. A. Sequence of phage-displayed scFv, A10, isolated from the one scFv library (V_L_ -linker-V_H_) [52] against biotinylated α1 subunit peptide. B. Sequence alignment of phage displayed scFvs isolated from a second library (V_H_ -linker-V_L_) [56] against biotinylated β2 (scFvs: A7, G8 and G11) subunit peptide.
(TIF)

Figure S2 **Expression of scFvs in** ***P. pastoris*** **.** A. 12% SDS-PAGE gel resolution of scFvs expressed and purified from *P. pastoris* supernatants. B. Binding of *Pichia*-expressed anti-GABA_A_ scFvs. Equal amounts of biotinylated target peptides or non-target peptide (negative control) were captured on NeutrAvidin™ (NA) coated microtiter plate wells, and after washing the binding of equivalent amounts of purified scFv protein, was monitored by ELISA. A biotinylated anti-Flag antibody was used to normalize the amounts of scFv added to each well. Error bars correspond to the standard deviation of triplicate measurements of the optical density of the wells at 405 nm wavelength. A7, G8 and G11 are anti-β2 binders while A10 is the anti-α1 binder.
(TIF)

Figure S3 **Binding of pre-absorbed scFvs at GABA_A_ receptors expressed in** ***Xenopus*** **oocytes.** Immunofluorescence of GABA_A_ receptor-expressing oocytes exposed to anti-α1 (A10) and anti-β2 (A7, G8, G11) scFvs that were pre-incubated with the cognate peptide overnight at 4°C. PC: positive control; GABA_A_ -expressing oocyte incubated with A10. Scale bar denotes 80 µm. A composite image of one of two (anti-α1) and three (anti-β2) independent trials is shown. A faint signal can be seen in the image of the GABA_A_-expressing oocyte treated with G8 that had been pre-incubated with its cognate peptide. We interpret this faint signal as to be due to non-zero background fluorescence.
(PDF)

Table S1 **Typical scFv yields from** ***P. pastoris*** **.** The best binding scFvs, which were isolated by phage-display, were expressed in *P. pastoris*, and their predicted size and yields are shown.
(PDF)
