# Supplementary figures and images for: Generation of Recombinant Antibodies to Rat GABAA Receptor Subunits by Affinity Selection on Synthetic Peptides
Source: PLoS One. 2014 Feb 19;9(2):e87964. doi: 10.1371/journal.pone.0087964 (PMC3929611; doi:10.1371/journal.pone.0087964)

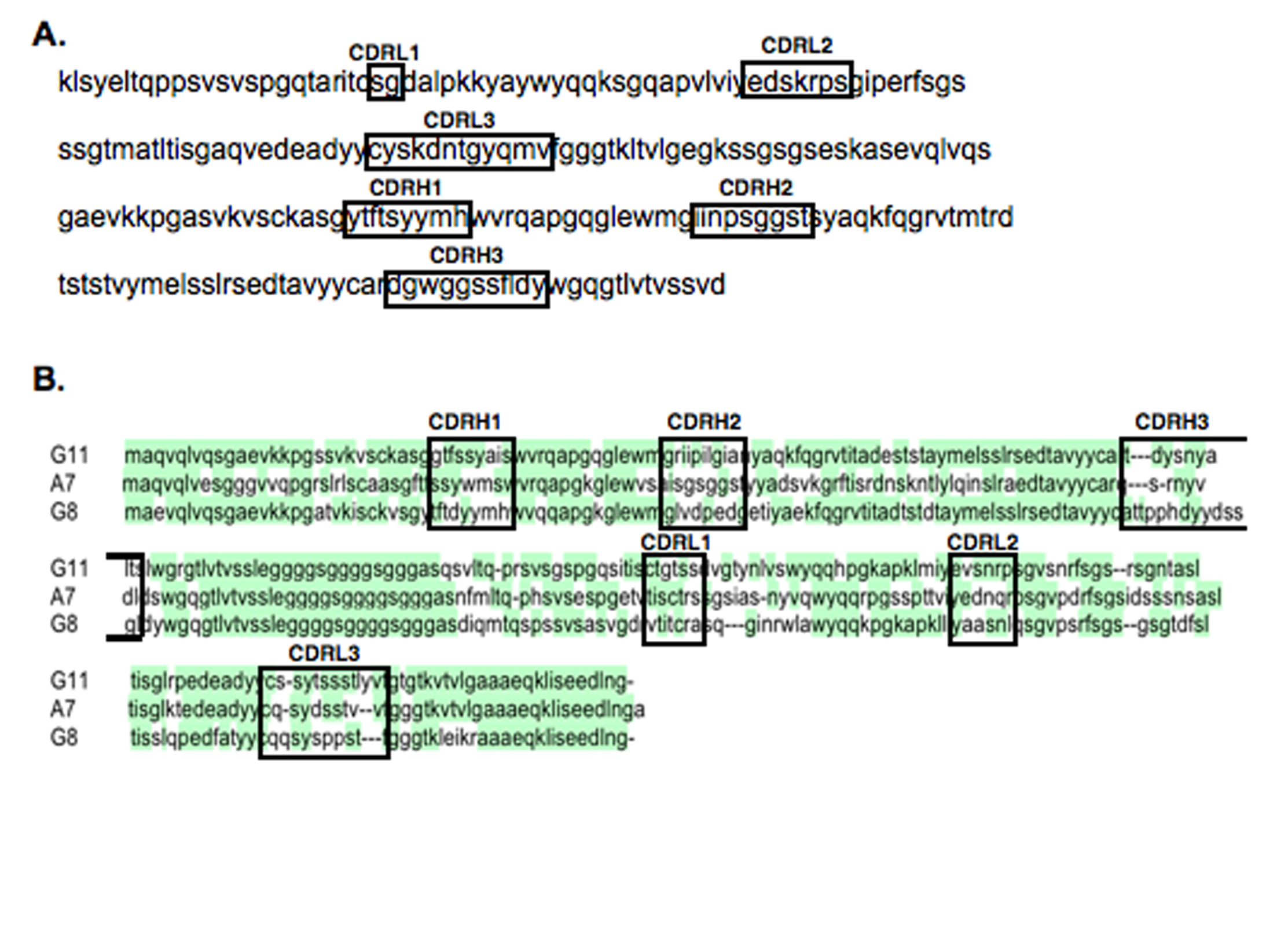

Supplement: Figure S1 — Sequence alignment of the predicted primary structures of anti-GABAA receptor subunit scFvs. Alignments of the primary structures of the scFv light (L) and heavy (H) chains and their complementarity determining regions, CDR, (boxes) were generated with Clone Manager (SciEd software). Regions of high similarity (>65%) are highlighted in light green. A. Sequence of phage-displayed scFv, A10, isolated from the one scFv library (VL -linker-VH) [52] against biotinylated α1 subunit peptide. B. Sequence alignment of phage displayed scFvs isolated from a second library (VH -linker-VL) [56] against biotinylated β2 (scFvs: A7, G8 and G11) subunit peptide. (TIF) [file pone.0087964.s001.tif]

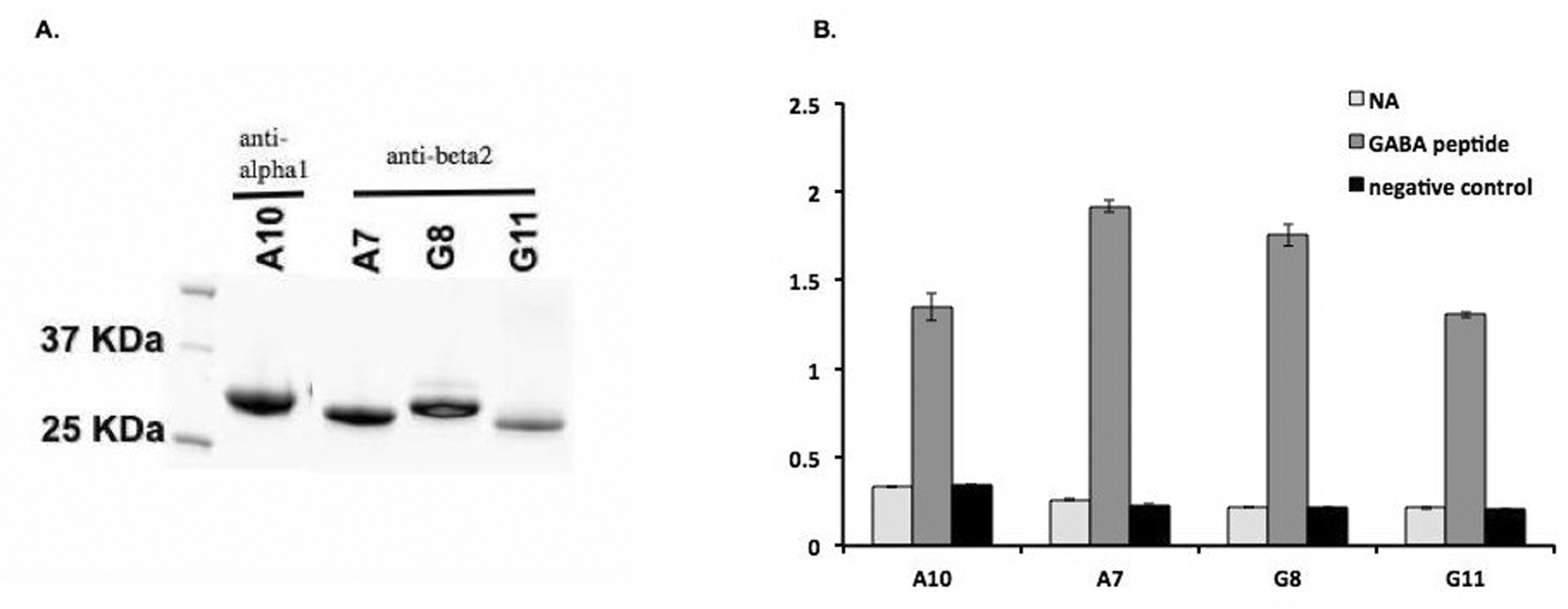

Supplement: Figure S2 — Expression of scFvs in P. pastoris . A. 12% SDS-PAGE gel resolution of scFvs expressed and purified from P. pastoris supernatants. B. Binding of Pichia-expressed anti-GABAA scFvs. Equal amounts of biotinylated target peptides or non-target peptide (negative control) were captured on NeutrAvidin™ (NA) coated microtiter plate wells, and after washing the binding of equivalent amounts of purified scFv protein, was monitored by ELISA. A biotinylated anti-Flag antibody was used to normalize the amounts of scFv added to each well. Error bars correspond to the standard deviation of triplicate measurements of the optical density of the wells at 405 nm wavelength. A7, G8 and G11 are anti-β2 binders while A10 is the anti-α1 binder. (TIF) [file pone.0087964.s002.tif]

Supplemental Figure S3:

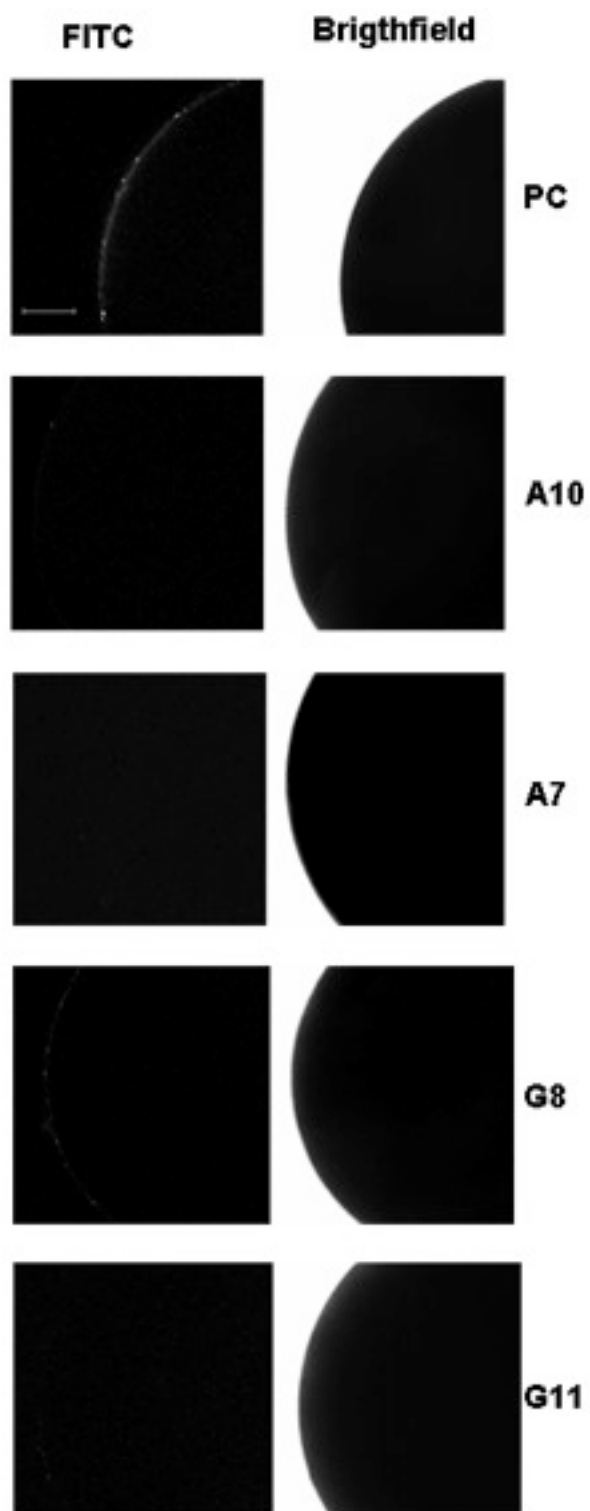

Supplement: Figure S3 — Binding of pre-absorbed scFvs at GABAA receptors expressed in Xenopus oocytes. Immunofluorescence of GABAA receptor-expressing oocytes exposed to anti-α1 (A10) and anti-β2 (A7, G8, G11) scFvs that were pre-incubated with the cognate peptide overnight at 4°C. PC: positive control; GABAA -expressing oocyte incubated with A10. Scale bar denotes 80 µm. A composite image of one of two (anti-α1) and three (anti-β2) independent trials is shown. A faint signal can be seen in the image of the GABAA-expressing oocyte treated with G8 that had been pre-incubated with its cognate peptide. We interpret this faint signal as to be due to non-zero background fluorescence. (PDF) [file pone.0087964.s003.pdf]
