# Supplementary material for: Generation of Recombinant Antibodies to Rat GABAA Receptor Subunits by Affinity Selection on Synthetic Peptides
Source: PLoS One. 2014 Feb 19;9(2):e87964. doi: 10.1371/journal.pone.0087964 (PMC3929611; doi:10.1371/journal.pone.0087964)
Supplement: Table S1 — Typical scFv yields from P. pastoris . The best binding scFvs, which were isolated by phage-display, were expressed in P. pastoris, and their predicted size and yields are shown. (PDF) [file pone.0087964.s004.pdf]

Supplemental Table S1: **Typical scFv yields from *P. pastoris*.**

	scFv	Molecular Weight (KDa)	Yield (mg/L)
anti-β2	G11	29.3	4
	G8	29.7	16.9
	A7	29.3	22.4
anti-α1	A10	28.5	11.9

The best binding scFvs, which were isolated by phage-display, were expressed in *P. pastoris*, and their predicted size and yields are shown.
